# Proteomic analysis of breast cancer based on immune subtypes

**DOI:** 10.1186/s12014-024-09463-y

**Published:** 2024-02-29

**Authors:** Yeonjin Jeon, GunHee Lee, Hwangkyo Jeong, Gyungyub Gong, JiSun Kim, Kyunggon Kim, Jae Ho Jeong, Hee Jin Lee

**Affiliations:** 1 Department of Pathology, Asan Medical Center, University of Ulsan College of Medicine, Seoul, Korea; 2 Prometabio Research Institute, Prometabio co., ltd, Hanam-Si, Gyeonggi-Do Republic of Korea; 3 Division of Breast Surgery, Department of Surgery, Asan Medical Center, University of Ulsan College of Medicine, Seoul, Korea; 4 Department of Digital Medicine, University of Ulsan College of Medicine, Seoul, Korea; 5 Department of Oncology, Asan Medical Center, University of Ulsan College of Medicine, Seoul, Korea; 6 Biomedical Sciences, Asan Medical Institute of Convergence Science and Technology (AMIST), Asan Medical Center, University of Ulsan College of Medicine, Seoul, Korea; 7 NeogenTC Corp, Seoul, Korea

**Keywords:** Breast cancer, Immune subtype, Proteomic analysis

## Abstract

**Background:**

Immunotherapy is applied to breast cancer to resolve the limitations of survival gain in existing treatment modalities. With immunotherapy, a tumor can be classified into immune-inflamed, excluded and desert based on the distribution of immune cells. We assessed the clinicopathological features, each subtype’s prognostic value and differentially expressed proteins between immune subtypes.

**Methods:**

Immune subtyping and proteomic analysis were performed on 56 breast cancer cases with neoadjuvant chemotherapy. The immune subtyping was based on the level of tumor-infiltrating lymphocytes (TILs) and Klintrup criteria. If the level of TILs was ≥ 10%, it was classified as immune-inflamed type without consideration of the Klintrup criteria. In cases of 1–9% TIL, Klintrup criteria 1–3 were classified as the immune-excluded subtype and Klintrup criteria not available (NA) was classified as NA. Cases of 1% TILs and Klintrup 0 were classified as the immune-desert subtype. Mass spectrometry was used to identify differentially expressed proteins in formalin-fixed paraffin-embedded biopsy tissues.

**Results:**

Of the 56 cases, 31 (55%) were immune-inflamed, 21 (38%) were immune-excluded, 2 (4%) were immune-desert and 2 (4%) were NA. Welch’s t-test revealed two differentially expressed proteins between immune-inflamed and immune-excluded/desert subtypes. Coronin-1A was upregulated in immune-inflamed tumors (adjusted *p* = 0.008) and α-1-antitrypsin was upregulated in immune-excluded/desert tumors (adjusted *p* = 0.008). Titin was upregulated in pathologic complete response (pCR) than non-pCR among immune-inflamed tumors (adjusted *p* = 0.036).

**Conclusions:**

Coronin-1A and α-1-antitrypsin were upregulated in immune-inflamed and immune-excluded/desert subtypes, respectively. Titin's elevated expression in pCR within the immune-inflamed subtype may indicate a favorable prognosis. Further studies involving large representative cohorts are necessary to validate these findings.

**Supplementary Information:**

The online version contains supplementary material available at 10.1186/s12014-024-09463-y.

## Background

Breast cancer accounts for a quarter of female cancers and has four intrinsic subtypes (luminal A, luminal B, HER2 positive and triple-negative) based on the status of the hormone receptor (HR) and human epithelial growth factor receptor 2 (HER2) [1, 2]. Metastatic triple-negative breast cancer (TNBC), which lacks a targetable antigen, has a median overall survival of only 13.3 months [3]. Immunotherapy has given hope for metastatic TNBC overexpressing programmed death-ligand 1 (PD-L1). Recently, the application of immunotherapy has also been extended to early and advanced breast cancer [4].

Immunotherapy employs the ability of endogenous T cells to recognize and kill tumor cells. To enhance the effectiveness of immunotherapy, T cells within tumor microenvironment (TME) should be recognized. Based on the spatial distribution of CD8 + T cells in TME, tumors can be classified into three immune subtypes, namely, immune-inflamed, immune-excluded and immune-desert subtypes. Immune-inflamed tumors are also called hot tumors, and have dense CD8 + T-cell infiltration, high level of tumor-infiltrating lymphocytes (TILs), high PD-L1 expression and high tumor mutation burden (TMB) [5]. Immune-excluded tumors have CD8 + T cells clustered at the tumor boundary but fail to infiltrate the tumor because of the immune-suppressed TME despite a high TMB. Immune-desert tumors have no aggregation of CD8 + T cells and are genomically stable and very proliferative. Immune-excluded and immune-desert tumors are also called cold tumors or non-inflamed tumors and have low PD-L1 expression, indicating that immunotherapy is limited and another treatment modality is needed [6].

We investigated the differences in clinicopathological features, prognosis and differentially expressed protein between immune subtypes in breast cancer. To the best of our knowledge, no study has defined immune subtypes by specific pathologic criteria; thus, we determined to define immune subtypes according to the level of TILs and inflammatory cell infiltrates (Klintrup criteria). Additionally, the tertiary lymphoid structure (TLS), which was known to be related to a better overall survival rate in breast cancer, was assessed [7]. All intrinsic types of breast cancer were included, allowing comparison between intrinsic subtypes. The study was conducted with patients who received neoadjuvant chemotherapy (NAC), allowing for independent evaluation of chemotherapeutic effects besides prognostic evaluation.

## Materials and methods

### Patients and clinical data

A total of 56 patients with breast cancer who received NAC and surgery at Asan Medical Center between 2014 and 2018 were examined. All patients were diagnosed with breast cancer by pre-NAC needle biopsy of the lesion and formalin-fixed paraffin-embedded (FFPE) samples were collected. Two FFPE samples were collected from one patient. Surgical therapy was either a breast-conserving operation or mastectomy and the surgical specimen was examined for residual tumor. Clinical features and follow-up data were collected from the medical records. The study protocol was approved by the Institutional Review Board of Asan Medical Center (2019–1480).

### Pathology data

**Fig. 1:**
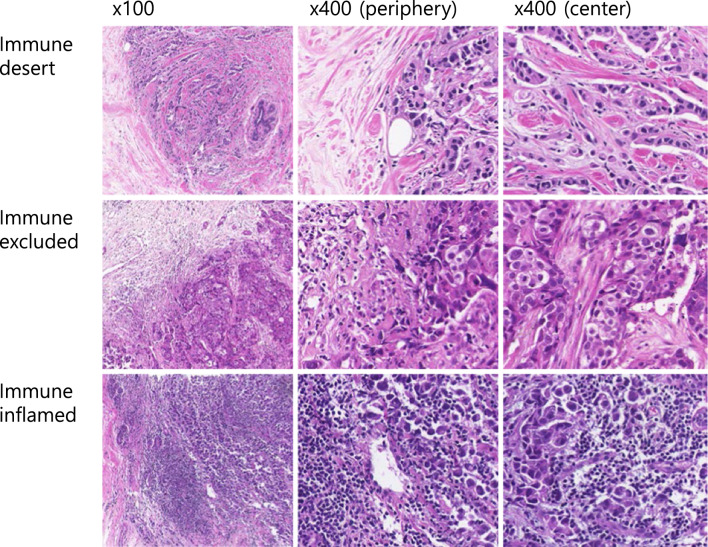
Representative images of immune subtypes

All pathologic features including intrinsic subtypes and histologic types were evaluated in the pre-NAC needle biopsy. All hematoxylin and eosin (H&E) slides were scanned on a PANNORAMIC 250 Flash III (3DHISTECH, Budapest, Hungary) with PANNORAMIC Scanner Software (3DHISTECH, Budapest, Hungary). Intrinsic subtypes were defined using immunostains or silver in situ hybridization tests of HR and HER2 as HR + HER2 − , HR + HER2 + , HR − HER2 + and TNBC [8]. The level of TILs was computed using the percentage area occupied by lymphoplasmacytic infiltration in the intra-tumoral stromal area. TLS, an ectopic lymph node-like structure in the peritumoral area was scored as 0, none; 1, minimal; 2, moderate and 3, marked. According to the Klintrup criteria, peritumoral inflammatory cell infiltration was graded as 0, none; 1, mild and patchy inflammatory cells; 2, prominent band-like inflammatory reaction and 3, florid cup-like inflammatory infiltrate [9]. As for the TLS and Klintrup criteria, if the specimen did not include the peritumoral area, it was classed as not available (NA). Immune subtypes were classified as desert (few inflammatory cell infiltration in the invasive tumor margin), excluded (inflammatory cell infiltration in only the invasive tumor margin and few at the intra-tumoral area) and inflamed (inflammatory cell infiltration intra-tumoral area through the invasive tumor margin) (Fig. 1). Specifically, if the level of TILs was ≥ 10%, it was classified as immune-inflamed without consideration of the Klintrup criteria. In cases of 1–9% TILs, Klintrup criteria 1–3 were classified as immune-excluded and Klintrup NA was classified as NA. Cases of 1% TILs and Klintrup 0 were classified as immune-desert (Table 1). Stroma (%) was characterized by the remaining area occupied by the invasive and in situ tumor in the intra-tumoral area and classified as low (< 50%) and high (≥ 50%). If no residual invasive tumor cell was discovered in the resected breast specimen following NAC, it was classified as pCR. According to the M.D. Anderson Cancer Center, residual cancer burden (RCB) class was computed from the primary tumor bed and lymph node status after NAC as 0 (pCR), I (minimal), II (moderate) and III (extensive) [10]. Relapse includes tumor recurrence and metastasis after surgical treatment.

**Table 1:** Immune subtyping using TILs (%) and the Klintrup criteria

TILs (%)	Klintrup criteria	Immune subtypes
1	0	Desert
1–9	1–3	Excluded
1–9	NA	NA
≥ 10	any	Inflamed

*TILs* tumor-infiltrating lymphocytes

### The cancer genome atlas (TCGA) data

R version 4.1.0 (R Core Team, 2021) and UCSC Xena were employed to retrieve RSEM normalized RNA-sequencing data from 1097 breast invasive carcinoma (BRCA) samples from TCGA. A correlation test with *CD8A*, which is a representative marker of T-cell and *CORO1A*, *HLA-A*, *H2AFY* and *SERPINA1,* was conducted using Pearson correlation.

### Sample preparation and mass spectrometry

Only the tumor area, except for normal area, by microscopic examination in pre-NAC FFPE samples was used for mass spectrometry. However, a portion of the normal area could be included because resection of the tumor area from the original sample was not conducted by microdissection. For protein extraction, 57 FFPE samples with the tumor area were deparaffinized using 100% heptane. The samples were incubated in an extraction buffer at 100 ℃ for 20 min, followed by 80 ℃ for 4 h. For the extracted protein quantification, the Bradford approach was employed. The prementioned procedure was conducted using QProteome FFPE Tissue Kit (Qiagen, Hilden, Germany). Protein was digested to a peptide using S-TrapTM (ProtiFi, Long Island, NY, USA). The resulting peptide mixture was dried and reconstituted using SolA (0.1% formic acid). Digested tryptic peptides were separated using a Dionex UltiMate 3000 RSLCnano system (Thermo Fisher Scientific) to separate on an Acclaim™ Pepmap 100 C18 column (500 mm × 75 μm i.d., 3 μm, 100 Å) equipped with a C18 Pepmap trap column (20 mm × 100 μm i.d., 5 μm, 100 Å; Thermo Scientific, USA) over 200 min with 300 nL/min flow rate. Peptide separation was performed using a 0–48% gradient in a Sol B (100% acetonitrile, 0.1% formic acid and 5% DMSO) for 150 min at 50 °C. A Q Exactive™ Plus Hybrid Quadrupole-Orbitrap™ mass spectrometer with a nano-ESI source was used to acquire spectra data with a data-dependent mode with a full scan and 20 of data-dependent MS/MS scans. The full scan MS spectra was obtained from a 350 to 1800 m/z with a maximum injection time of 50 ms and a resolution of 70,000 at m/z 400 was used. The selected ions were fragmented by higher-energy collisional dissociation (HCD) using the following parameters: 1.7 Da precursor ion isolation window, 27% normalized collision energy with a maximum injection time of 100 ms and a resolution of 17,500 at m/z 400.

### Mass spectrum processing

The MS/MS spectra from LC–MS analysis were processed with the SequestHT algorithm embedded in Proteome Discoverer (version 2.4, Thermo Fisher Scientific) using the SwissProt human protein sequence database (March 2021). In details, precursor mass tolerance was set to ± 10 ppm and MS/MS tolerance was set at 0.02 Da. The search parameters were set as default including cysteine carbamidomethylation as a fixed modification and N-terminal acetylation and methionine oxidation as variable modifications with two miscleavages. The 1% of false discovery rates were set on peptide identification using “Percolator” module. For relative quantitation analysis, label-free quantitation was performed using the peak intensity for the unique and razor peptides of each protein. Abundance information of each protein was extracted for further statistical analysis. The mass spectrometry proteomics data have been deposited to the ProteomeXchange Consortium via the PRIDE partner repository with the dataset identifier PXD043902.

### Statistical analysis of proteome data

All statistical analysis was conducted in R version 4.1.0 (R Core Team, 2021). In raw data, 57 samples with 5014 proteins were discovered. Among them, the case with two samples was later replaced with one sample with more available data. Two cases with NA for immune subtyping due to the tumor boundary absence and TILs < 10% were removed. The protein with NA data from any samples was removed because imputation was not conducted in this investigation due to insufficient sample size. Therefore, 54 samples with 341 proteins were collected. To examine more proteins for further analysis, the two cases with the most NA proteins were eliminated. Thus, 855 proteins were collected. Using the R package preprocessCore, the original protein abundance value was Log2-transformed and quantile normalized. Gene Ontology (GO) enrichment analysis was performed using ToppGene after normalization. The source of substantially enriched pathways was collected from ToppGene databases. In immune-inflamed vs. immune-excluded and immune-desert subtypes, pCR vs. non-pCR in the total cohort and pCR vs. non-PCR in the immune-inflamed subtype, Welch’s t-test was performed to compare means between the two groups. A volcano plot was employed to visualize the fold change and p-value. For proteins with adjusted *p* < 0.05, heatmap and violin plots were generated.

### Statistical analysis of clinical and pathology data

All statistical analyses were performed using IBM SPSS Statistics for Windows version 20.0 (IBM Corp., Armonk, NY, USA). The χ^2^ test was employed to compute correlations between immune subtypes and clinicopathologic features. Log-rank tests and Kaplan–Meier (KM) survival curves were employed to evaluate differences between the immune subtypes. Overall survival (OS) and relapse-free survival (RFS) were evaluated using the length of time (months) from the date of surgical treatment to the date of death and relapse. Univariate regression analysis using the Cox proportional hazards model was employed to estimate the immune subtype’s hazard ratios and other clinicopathological features. *p* < 0.05 was considered statistically substantial.

## Results

### Clinicopathologic features and immune subtypes of breast cancer

All patients were female, with age ranging from 32 to 66 (median, 46) years. The most common intrinsic subtype was TNBC (n = 24, 43%), followed by HR + HER2 − (n = 20, 36%). The histologic type consisted of invasive breast carcinoma of no special type (n = 53, 95%) and invasive lobular carcinoma (n = 3, 5%). Two cases had bilateral breast tumors at the time of diagnosis. The bilateral tumors of each case had similar histologic and intrinsic subtypes and the evaluation of the clinicopathologic features of these cases was based on the sample employed for the proteomic analysis. The follow-up period ranged from 4.6 to 84.0 (median, 54.6) months. Relapse occurred in 18 (32%) cases and the most common relapse site was the bone (n = 9), followed by the lung (n = 6).

When classified according to the immune subtype, there were 2 (4%) cases of desert types, 21 (38%) of excluded types and 31 (55%) of inflamed types. Two cases with no peritumoral area and < 10% TILs were removed from the analysis because discriminating these cases as desert or excluded type was impossible. Two cases of bilateral tumors have similar immune subtypes. Immune-desert and immune-excluded subtypes were grouped for further evaluation because of the very few immune-desert cases. When the correlation between clinicopathologic features and immune subtype was examined, only age was substantially related to the immune subtypes (*p* = 0.039); the old age group (≥ 50) was more common in immune-inflamed than immune-excluded/desert subtypes. The pCR rate was higher in immune-inflamed (42%) than immune-excluded/desert (17%) subtypes, but that was not statistically different (*p* = 0.055) (Table 2).

**Table 2:** Correlation between the clinicopathologic features and immune subtypes in patients with breast cancer

Factors	Immune subtypes		*p*-value
	Inflamed (%)	Excluded/desert (%)	
Age			0.039*
< 50	21 (68)	21 (91)	
≥ 50	10 (32)	2 (9)	
Subtypes			0.066
HR + HER2−	7 (23)	11 (48)	
HR + HER2 +	4 (13)	2 (9)	
HR-HER2 +	2 (7)	4 (17)	
TNBC	18 (58)	6 (26)	
Histologic types			0.386
IBC-NST	30 (97)	21 (91)	
ILC	1 (3)	2 (9)	
Non-pCR/pCR			0.055
Non-pCR	18 (58)	19 (83)	
pCR	13 (42)	4 (17)	
RCB class			0.223
0	3 (10)	2 (9)	
I	11 (36)	14 (61)	
II	4 (13)	3 (13)	
III	13 (42)	4 (17)	
Non-relapse/Relapse			0.887
Non-relapse	21 (68)	16 (70)	
Relapse	10 (32)	7 (30)	
TILs (%)			< 0.001*
< 10	0	23 (100)	
10–19	12 (39)	0	
≥ 20	19 (61)	0	
TLS score			0.45
0	15 (52)	16 (70)	
1	6 (21)	4 (17)	
2	3 (10)	2 (9)	
3	5 (17)	1 (4)	
Klintrup criteria			< 0.001*
0	0	2 (9)	
1	3 (10)	14 (61)	
2	16 (55)	4 (17)	
3	10 (35)	3 (13)	
Stroma (%)			0.594
Low (< 50)	13 (42)	8 (35)	
High (≥ 50)	18 (58)	15 (65)	

*HR* hormone receptors, *HER2* human epithelial growth factor receptor 2, *IBC-NST* invasive breast carcinoma of no special type, *ILC* invasive lobular carcinoma, *pCR* pathologic complete response, *RCB* residual cancer burden, *TILs* tumor-infiltrating lymphocytes, *TLS* tertiary lymphoid structure *p < 0.05

### Prognostic value of immune subtypes in breast cancer

The KM survival analysis with a log-rank test was employed to examine the prognostic value of immune subtypes in breast cancer. No statistically significant difference was found between the three immune subtypes in OS (*p* = 0.666) and RFS (*p* = 0.795). Moreover, no statistically significant difference in OS (*p* = 0.440) and RFS (*p* = 0.776) was detected between immune-excluded/desert and immune-inflamed subtypes. Among intrinsic subtypes of breast cancer, TNBC had a poor prognosis [11]. For the exact evaluation of the prognostic value of immune subtypes without the effect of the intrinsic subtype, the equivalent test was performed on TNBC cases. However, no statistically significant change between immune subtypes was discovered (Additional file 1: Fig. S1). No statistically significant prognostic parameter for OS was noted when the univariate Cox model was employed to estimate the prognostic value of immune subtypes and other clinicopathological factors such as age, intrinsic subtypes, pCR rate, RCB class, TILs, Klintrup criteria, TLS score and stromal per cent. As for the RFS, pCR (vs. non-pCR, *p* = 0.045) and RCB class 0 (vs. III, *p* = 0.038) were good prognostic parameters (Table 3).

**Table 3:** Univariate analysis comparing OS and RFS in breast cancer

Factors	OS	*p*	RFS	*p*
	Hazard Ratio (95% CI)		Hazard Ratio (95% CI)	
Age (≥ 50 vs. < 50)	1.990 (0.495–7.999)	0.332	1.475 (0.523–4.161)	0.463
Intrinsic subtypes				
HR + HER2- (ref.)	1		1	
HR + HER2 +	3.572 (0.223–57.127)	0.368	0.593 (0.071–4.942)	0.629
HR− HER2 +	0.000	−	0.542 (0.065–4.519)	0.572
TNBC	7.422 (0.911–60.480)	0.061	1.712 (0.620–4.727)	0.299
pCR vs. non-pCR	0.026 (0.000–8.589)	0.217	0.221 (0.050–0.964)	0.045*
RCB class				
0	0.000	−	0.166 (0.030–0.909)	0.038*
I	0.000	−	0.000	−
II	1.009 (0.209–4.859)	0.991	0.838 (0.270–2.604)	0.760
III (ref.)	1		1	
Immune subtypes				
Excluded/Desert (ref.)	1		1	
Inflamed	1.716 (0.428–6.882)	0.446	1.150 (0.437–3.027)	0.777
TILs (≥ 10% vs. < 10%)	1.872 (0.467–7.505)	0.376	1.110 (0.437–2.819)	0.826
TILs (%)	1.019 (0.988–1.051)	0.242	1.006 (0.978–1.034)	0.690
Klintrup criteria (2,3 vs. 0,1)	2.393 (0.496–11.543)	0.277	1.175 (0.434–3.182)	0.752
TLS score (2,3 vs. 0,1)	3.360 (0.900–12.544)	0.071	2.494 (0.920–6.761)	0.073
Stroma (≥ 50% vs. < 50%)	0.495 (0.133–1.845)	0.295	0.742 (0.293–1.881)	0.530

*OS* overall survival, *RFS* relapse-free survival, *CI* confidence interval, *p p*-value, *pCR* pathologic complete response, *RCB* residual cancer burden, *TILs* tumor-infiltrating lymphocytes, *TLS* tertial lymphoid structure *p < 0.05

### Analysis of proteomic data

Among 5014 proteins discovered initially in 57 samples, two cases with most NA data (F53 and F54) and two cases with NA immune subtype (F06 and F50) were eliminated. One case containing two samples (F39 and F45) was performed further with a sample (F39), which had more available data. Finally, for proteomic analysis, 855 proteins with 52 cases were employed (Additional file 2: Fig. S2). Additional file 3: Table shows the gene-enrichment analysis of raw data (5014 proteins) and confirmed data (855 proteins), with an adjusted *p* < 0.05. Inferring what kinds of proteins were readily extracted after the formalin fixation procedure of breast cancer using factors listed in the Additional file 3: Table was possible.

### Proteomic differential expressions between immune subtypes

**Fig. 2:**
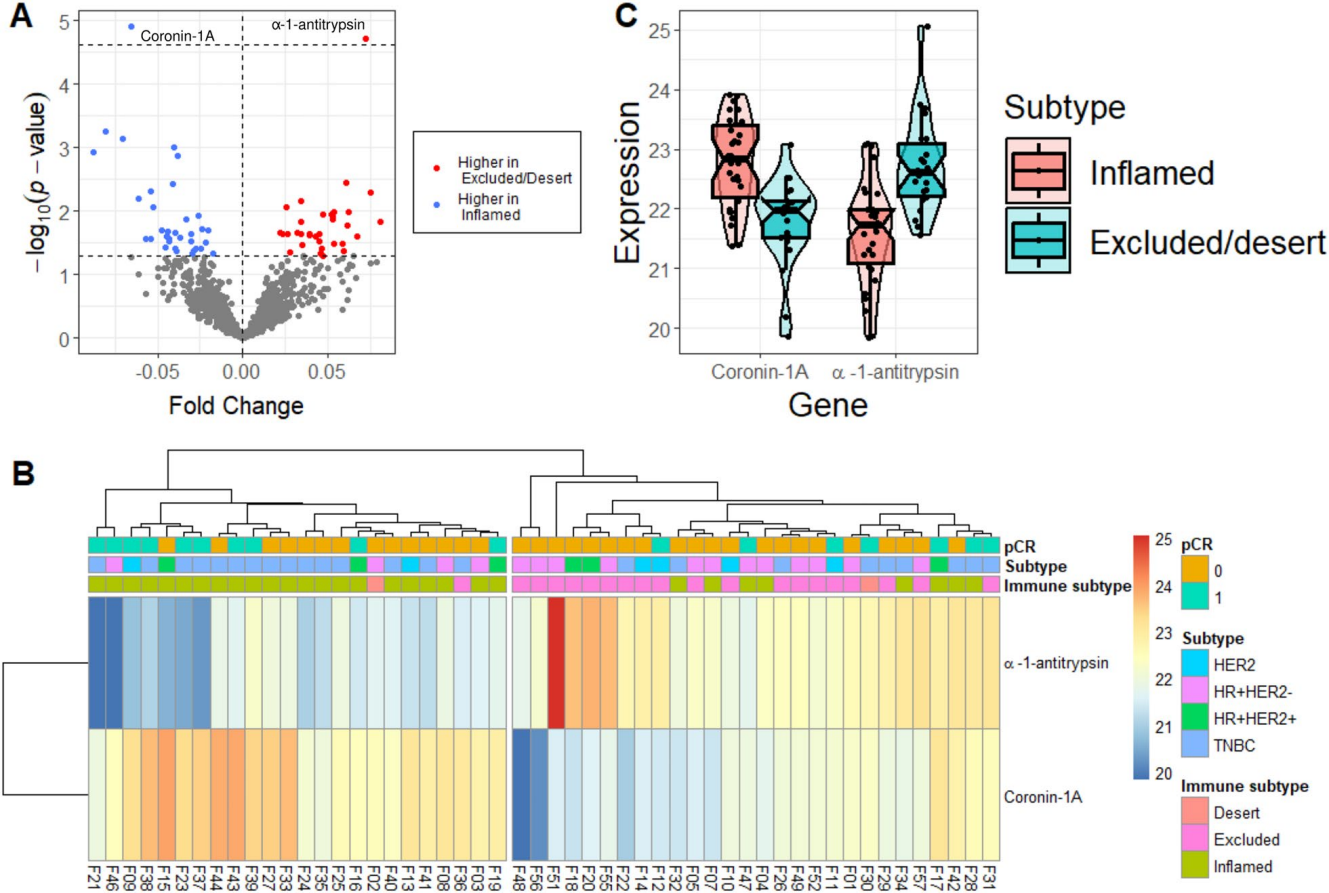
Differential proteomics expressions between immune subtypes. **a** The volcano plot shows the fold change between the immune-inflamed and immune-excluded/desert subtypes. **b** Heatmap for α-1-antitrypsin and coronin-1A (cutree = 2). **c** Violin plots for coronin-1A and α-1-antitrypsin in the immune-inflamed and immune-excluded/desert subtypes

Welch’s t-test was conducted to investigate a change in proteomic expression between immune-inflamed and immune-excluded/desert subtypes. Figure 2a shows a volcano plot representing the fold change (immune-excluded/desert / immune-inflamed) in expression and *p*-value. Among 855 proteins that had no NA data for all samples, two proteins showed statistically significant differential expression. Coronin-1A (*CORO1A*) and serpin family A member 1 (*SERPINA1*, α-1-antitrypsin) were upregulated in the immune-inflamed (adjusted *p* = 0.008) and immune-excluded/desert (adjusted *p* = 0.008), subtypes, respectively. Heatmap (cutree = 2) (Fig. 2b) and violin plots (Fig. 2c) show two proteins with a similar pattern. Coronin-1A in immune-inflamed and α-1-antitrypsin in immune-excluded/desert groups were both upregulated.

### Correlation of protein expression with the TIL level

**Fig. 3:**
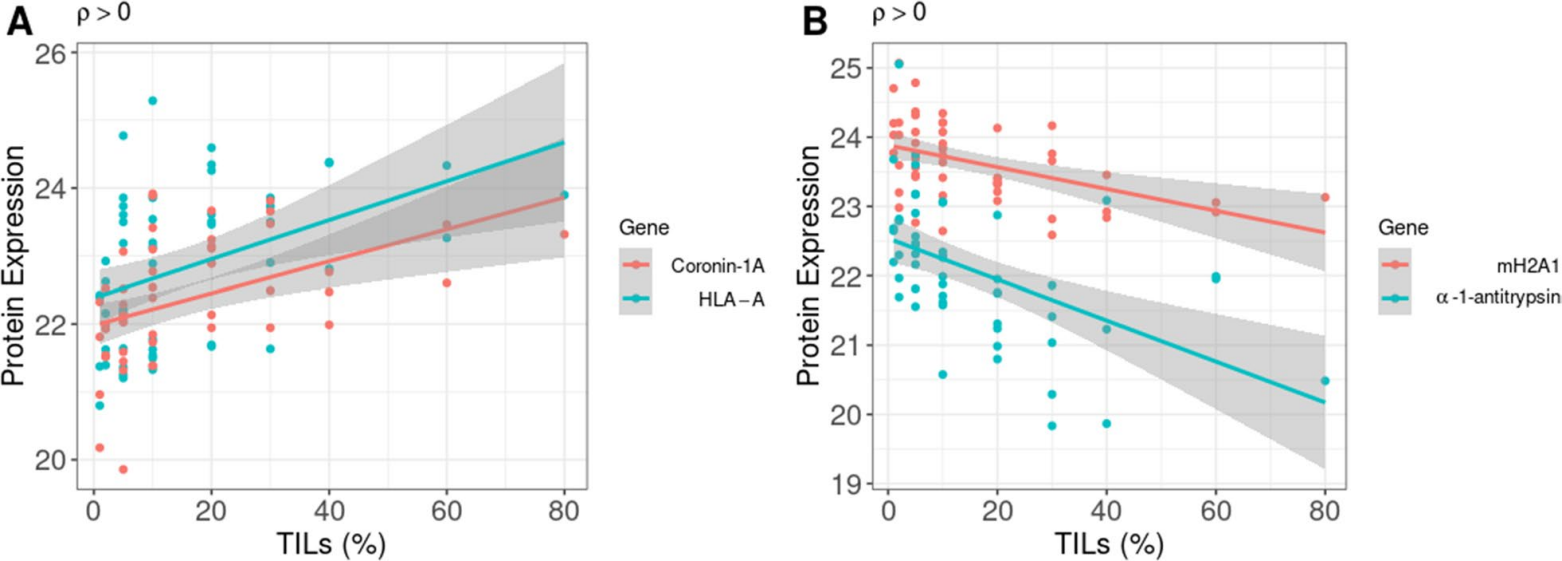
Correlation between protein expression and the TIL level. **a** Positively correlated proteins. **b** Negatively correlated proteins

In this study, immune subtype’s designation was largely dependent on the criteria of 10% of TILs. We used Spearman’s correlation to investigate the correlation between protein expression and TIL level (as continuous variables). Four proteins with adjusted *p* < 0.05 were discovered. Coronin-1A and HLA-A (*HLA-A*) positively correlated with TILs. mH2A1 (*H2AFY*) and α-1-antitrypsin negatively correlated with TILs (Fig. 3). Coronin-1A and α-1-antitrypsin were discovered in the positively and negatively correlated groups, respectively. We conducted a Pearson correlation using RNA-seq from 1,097 TCGA BRCA samples in 4 proteins with *CD8A* (T-cell marker) to confirm this finding. As expected, *CORO1A* (r = 0.729, *p* < 0.001) and *HLA-A* (r = 0.485, *p* < 0.001) positively correlated with *CD8A* and *H2AFY* (r =  − 0.104, *p* < 0.001) negatively correlated with *CD8A*. In contrast to our finding, *SERPINA1* positively correlated with *CD8A* (r = 0.114, *p* < 0.001).

### Proteomic differential expressions between NAC responses

**Fig. 4:**
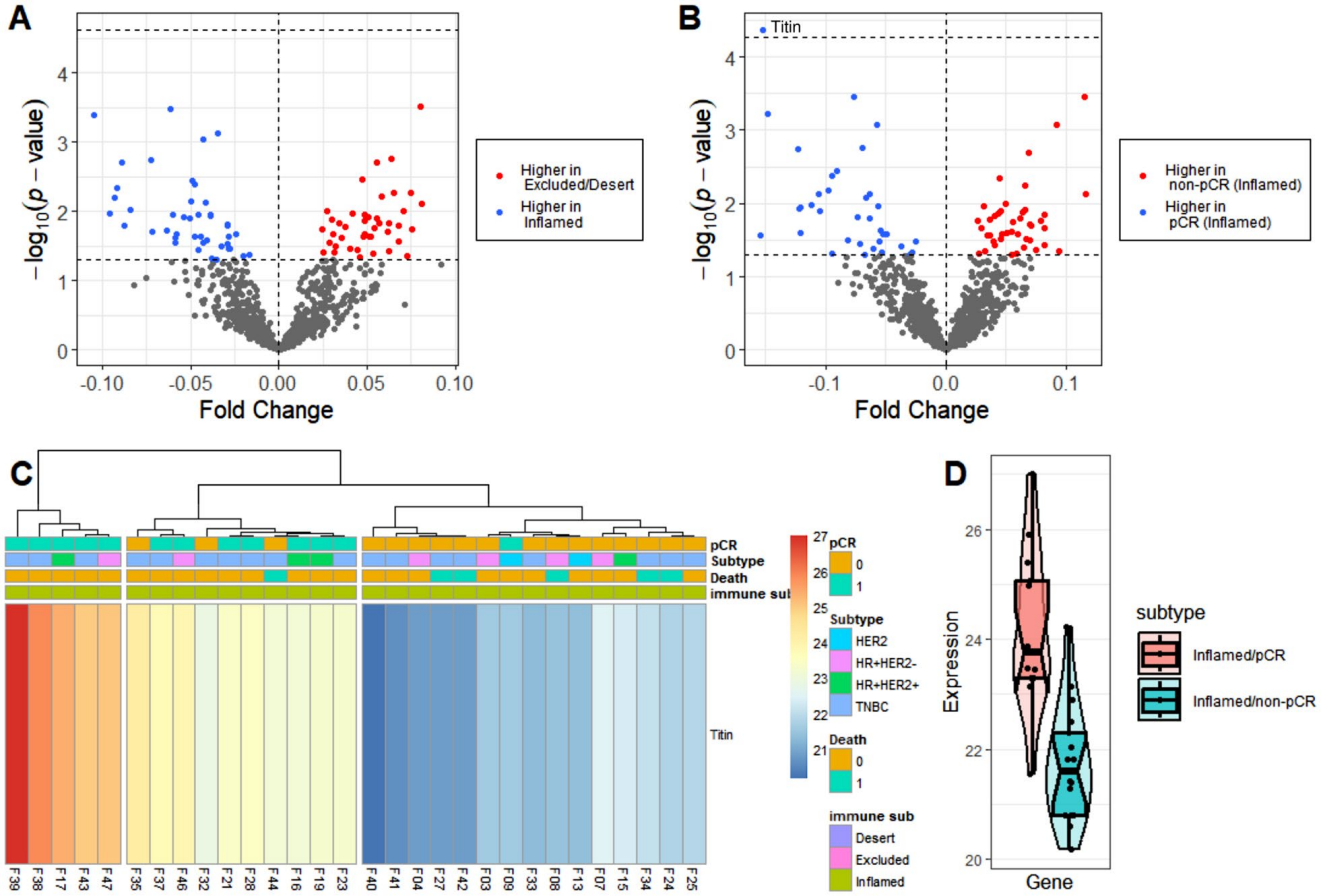
Proteomic differential expressions between NAC responses. **a** The volcano plot shows the fold change between pCR and non-pCR in the entire cohort. **b** Volcano plot shows the fold change between pCR and non-pCR in the immune-inflamed subtype. **c** Heatmap for titin (cutree = 3). **d** Violin plots for titin in the immune-inflamed subtype

Furthermore, Welch’s t-test was conducted to identify the difference between pCR and non-pCR in the entire cohort. The volcano plot showing fold change (non-pCR/pCR) in the expression showed no statistically difference between the two groups (Fig. 4a). The immune-inflamed subtypes, which contained high TILs, were discovered to have a high pCR rate after NAC for breast cancer. In our study, 58% of the immune-inflamed subtype contained non-PCR after NAC. Welch’s test between pCR and non-pCR was conducted to investigate the inhibitory factors of NAC in non-pCR cases with immune-inflamed phenotypes. In non-pCR, no protein was statistically substantially upregulated. Titin was the only protein upregulated in pCR (adjusted *p* = 0.036) (Fig. 4b). For titin, the heatmap (Fig. 4c) and violin plots (Fig. 4d) in the immune-inflamed subtype also showed upregulation in the pCR group.

## Discussion

To overcome the limitations in the survival gain of pre-existing therapies to conquer breast cancer, immunotherapy which inhibits the immunosuppressive effect of tumor cells was implemented [12]. To improve the treatment effects of immunotherapy and discover a new target, molecular studies are necessary. Proteins were thought to reflect functional tumoral biology more directly than genes or transcripts. In addition, a study showed a copy number of protein, and mRNA was not completely correlated. Therefore, proteomic studies are necessary for the accurate evaluation of tumor biology [13]. Proteomic analysis was conducted in breast cancer for different goals, such as identifying candidate immunotherapeutic targets and proteomic patterns related to NAC responses [14, 15]. Our study performed a proteomic analysis of breast cancer including all intrinsic subtypes with NAC to offer the opportunity for new treatment development.

In previous studies, the immune-inflamed subtype was discovered to have a better response to immune checkpoint inhibitors than immune-excluded or immune-desert subtypes [16, 17]. We assessed the relationship between immune subtypes and other clinicopathological factors. The immune-inflamed subtype had a higher pCR rate than immune-excluded/desert subtypes; however, it was not statistically significant (*p* = 0.055). Other factors such as intrinsic subtypes, histologic subtypes, RCB class, relapse rate, TLS score and percentage of stroma did not show a statistically significant difference. Only old age was statistically significantly more common in immune-inflamed than in immune-excluded/desert subtypes because of different TNBC prevalence in each age group. TNBC in old age was 6 (50%) cases, on the other hand, TNBC in the young age was 18 (41%) cases. In this study, TNBC mostly consisted of immune-inflamed subtypes (n = 18, 75%), which was consistent with the results of a previous study [18].

In evaluating the prognostic value of immune subtypes, OS and RFS showed no significant difference in the three-tier (inflamed, excluded and desert) or two-tier (inflamed and excluded/desert) classification using KM survival curves. The percentage of TNBC in all breast cancers was approximately 10%; however, in the present study, TNBC accounted for nearly half of the cohort (n = 24, 43%) [19]. Similarly, no statistical significance in OS and RFS was found in the TNBC cohort alone. In the univariate Cox hazards model for OS and RFS, immune subtypes and other factors such as age, intrinsic subtypes, TILs, Klintrup criteria, TLS score and stromal percentage were not statistically significant prognostic OS parameters. Only pCR (vs. non-pCR) and RCB class 0 (vs. III) were demonstrated as good prognostic factors. Although some studies have shown that the presence of TLS in breast cancer and high TILs level in TNBC were good prognostic factors, the presence of TLS and high TIL levels were not significant prognostic factors in our study [7, 20].

In this study, three proteins indicated differential expressions in the proteomic analysis. Coronin-1A, encoded by *CORO1A*, was upregulated in immune-inflamed than in immune-excluded/desert subtypes. The upregulation of coronin-1A might be associated with a good response to immunotherapy. Similarly, a recent study showed that *CORO1A* in ductal breast tumors was overexpressed in immune-inflamed subtypes in the Molecular Taxonomy of Breast Cancer International Consortium (METABRIC) and TCGA data by whole transcriptome analysis [21]. Another study demonstrated that *CORO1A* was overexpressed in TNBC and a good prognostic factor in TCGA RNA-seq data [22]. *CORO1A* upregulation in immune-inflamed subtypes might be associated with the fact that *CORO1A* was dominantly expressed in leukocytes and engaged in the use of macrophage, T-cell receptor signaling and lymphocyte trafficking [23]. However, the precise relationship between *CORO1A* and tumor biology will be revealed in further studies.

α-1-antitrypsin, an extracellular matrix protein, encoded by *SERPINA1*, was upregulated in immune-excluded/desert than in immune-inflamed subtypes. The upregulation of α-1-antitrypsin might be associated with an unfavorable response to immunotherapy. A study indicated that α-1-antitrypsin overexpression improves the migration of breast cancer cells using migration assay and high α-1-antitrypsin immunohistochemical expression has unfavorable prognostic value in patients with colorectal carcinoma [24]. Conversely, other investigations have found that high *SERPINA1* expression had a good prognosis in breast cancer based on TCGA gene expression data [25, 26]. A high *SEPINA1* expression has a good prognostic value based on TCGA gene expression data in estrogen receptor (ER) + HER2 + breast cancer, not in ER- or ER + HER2- breast cancer, according to Chan et el. [27]. Furthermore, when *SEPINA1* expression was divided into high and low by mean in this study, the KM survival analysis revealed no OS difference between the high and low *SERPINA1* expression groups (*p* = 0.785), which was possible given the variable distributions of intrinsic types of breast cancer. The evaluation of prognostic value was limited because only one of six patients with ER + HER2 + breast cancer died in this cohort. Since there were conflicting points about α-1-antitrypsin’s influence on cancer cell biology and prognostic value, the application of α-1-antitrypsin as a therapeutic target is limited.

Titin, encoded by *TTN*, was upregulated in pCR than in non-pCR in the immune-inflamed subtype. Titin could be related to a favorable prognosis. However, Lips et al. revealed that *TTN* was one of the most frequently observed alterations in TNBC and the mutation rate of *TTN* was similar between the chemotherapy response group and the non-response group by next-generation sequencing [28]. Kim et al. showed that *TTN* was frequently mutated but have no significant prognostic value in breast cancer [29]. The use of *TTN* as a good predictable factor for chemotherapy in breast cancer should be considered after revealing the role and clinical significance of *TTN* mutation in breast cancer in further studies.

By the Spearman correlation, coronin-1A and HLA-A were positively correlated with TILs and mH2A1 and α-1-antitrypsin were negatively correlated with TILs. *CD8A*, a marker of T-cell, was employed as a surrogate TIL marker for correlation analysis with four proteins from TCGA data because TILs consist of lymphoplasmacytic cells. The findings regarding *CORO1A*, *HLA-A* and *H2AFY* were similar to ours, but *SERPINA1* revealed a contrasting finding. This could be attributed to the different expression values of molecules between the proteomic analysis and the transcriptomic analysis. The proteomic expression level could be different from the mRNA level because the protein level is affected by post-transcriptional and post-translational regulations and protein half-lives [30]. As coronin-1A was enriched in immune-inflamed than immune-excluded/desert in our study and *CORO1A* was positively correlated with *CD8A* in TCGA data, coronin-1A might be used as a predictive response factor for immunotherapy.

This study had some limitations. Firstly, since the case selection was not consecutive but purposely included an equal number of cases for pCR and non-pCR, the distribution of the intrinsic subtypes of breast cancer was different from the known natural distribution. If the case selection was conducted consecutively, a representative study would have been possible. Secondly, 5,014 proteins were initially detected from mass spectrometry; however, several proteins have NA data. Therefore, 855 proteins were employed for further differential study. Numerous NA data might be due to the use of FFPE samples for proteomic analysis because formalin can induce a cross-link of proteins that interrupts protein extraction [31]. However, the use of FFPE samples employed routinely for tissue preservation in the proteomic analysis is crucial. Some studies of breast cancer have dealt with FFPE samples effectively using more than 2000 proteins for analysis [15, 32]. The various fixation times in this study might result in a low rate of protein identification because of the effect of the probability of fixation time on protein extraction caused by the degree of cross-link [33]. For a more exact and efficient proteomic analysis, fresh tissue or fixation time control should be used. Lastly, we investigated only pre-NAC tissue for proteomic analysis. If post-NAC or corresponding normal tissues were employed, protein changes during chemotherapy or specifically expressed proteins in the tumor could be discovered [15, 34].

## Conclusions

We investigated clinicopathologic features, prognostic values and type of protein expressed in each immune subtype of breast cancer using FFPE samples. We identified that coronin-1A was upregulated in the immune-inflamed subtype, α-1-antitrypsin was upregulated in the immune-excluded/desert subtype and titin was upregulated in pCR in the immune-inflamed subtype by proteomic analysis. Further studies involving a large, representative cohort must be conducted to confirm these findings.

### Supplementary Information

**Additional file 1: Figure S1.** Survival rate between immune subtypes in the total cohort and TNBC cohort. (**a**) OS in three immune subtypes (*p* = 0.666) in the total cohort. (**b**) OS in two immune groups in the total cohort (*p* = 0.440). (**c**) RFS in three immune subtypes in the total cohort (*p* = 0.795). (**d**) RFS in two immune groups in the total cohort (*p* = 0.776). (**e**) OS in three immune subtypes (*p* = 0.777) in the TNBC cohort. (**f**) OS in two immune groups in the TNBC cohort (p = 0.930). (**g**) RFS in three immune subtypes in the TNBC cohort (p = 0.630). (**h**) RFS in two immune groups in the TNBC cohort (p = 0.820).

**Additional file 2: Figure S2.** The number of identified proteins in the total cohort. Each column states the number of recognized proteins (blue) and NA proteins (red) of each sample among the initially recognized 5,014 proteins in the total cohort. The last column (yellow) states the number of proteins, which have verifiable data for all samples.

**Additional file 3: Table.** Gene-enrichment analysis of raw data (5,014 proteins) and confirmed data (855 proteins).

## Data Availability

The datasets used and/or analyzed during the current study are available from the corresponding author on reasonable request.

## Abbreviations

TILs: Tumor-infiltrating lymphocytes
NA: Not available
FFPE: Formalin-fixed paraffin-embedded
pCR: Pathologic complete response
HR: Hormone receptor
HER2: Human epithelial growth factor receptor 2
TNBC: Triple-negative breast cancer
PD-L1: Programmed death-ligand 1
TME: Tumor microenvironment
TMB: Tumor mutation burden
TLS: Tertiary lymphoid structure
NAC: Neoadjuvant chemotherapy
TCGA: The Cancer Genome Atlas
GO: Gene Ontology
KM: Kaplan–Meier
OS: Overall survival
RFS: Relapse-free survival
ER: Estrogen receptor
