# Supplementary material for: Proteomic analysis of breast cancer based on immune subtypes
Source: Clin Proteomics. 2024 Feb 29;21:17. doi: 10.1186/s12014-024-09463-y (PMC10905797; doi:10.1186/s12014-024-09463-y)
Supplement: Supplementary file 1 — Additional file 1: Figure S1. Survival rate between immune subtypes in the total cohort and TNBC cohort. (a) OS in three immune subtypes (p = 0.666) in the total cohort. (b) OS in two immune groups in the total cohort (p = 0.440). (c) RFS in three immune subtypes in the total cohort (p = 0.795). (d) RFS in two immune groups in the total cohort (p = 0.776). (e) OS in three immune subtypes (p = 0.777) in the TNBC cohort. (f) OS in two immune groups in the TNBC cohort (p = 0.930). (g) RFS in three immune subtypes in the TNBC cohort (p = 0.630). (h) RFS in two immune groups in the TNBC cohort (p = 0.820). [file 12014_2024_9463_MOESM1_ESM.docx]

**Additional file 1: Figure S1.** Survival rate between immune subtypes in the total cohort and TNBC cohort. (**a**) OS in three immune subtypes (*p*=.666) in the total cohort. (**b**) OS in two immune groups in the total cohort (*p*=.440). (**c**) RFS in three immune subtypes in the total cohort (*p*=.795). (**d**) RFS in two immune groups in the total cohort (*p*=.776). (**e**) OS in three immune subtypes (*p*=.777) in the TNBC cohort. (**f**) OS in two immune groups in the TNBC cohort (p=.930). (**g**) RFS in three immune subtypes in the TNBC cohort (p=.630). (**h**) RFS in two immune groups in the TNBC cohort (p=.820).


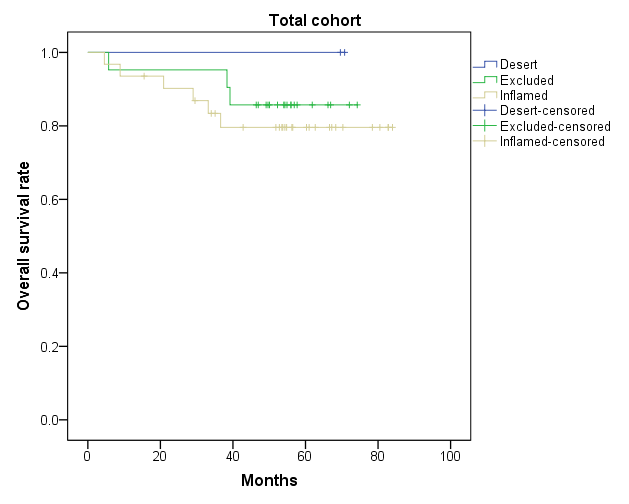

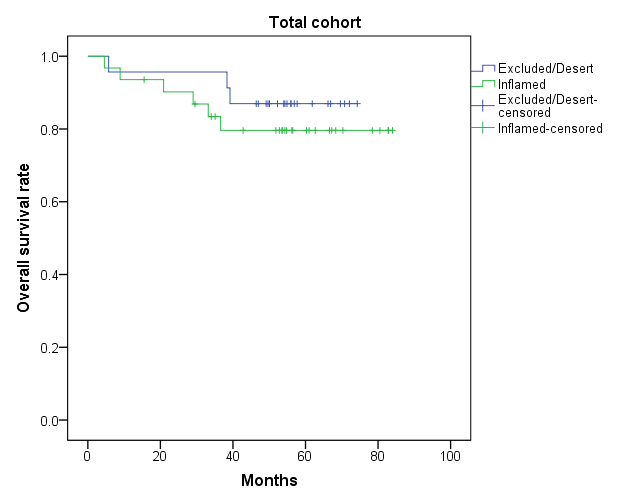

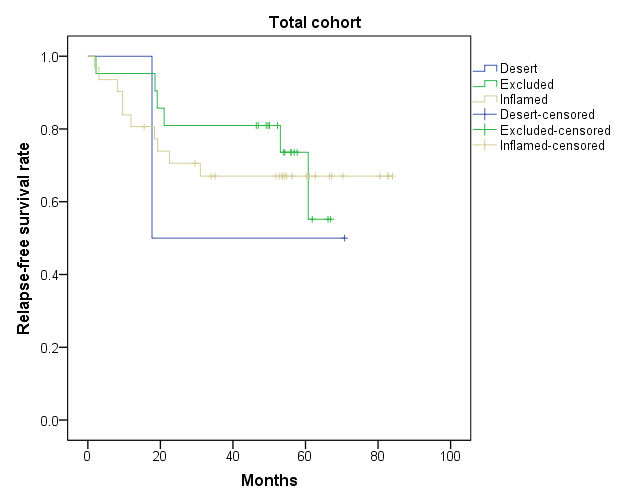

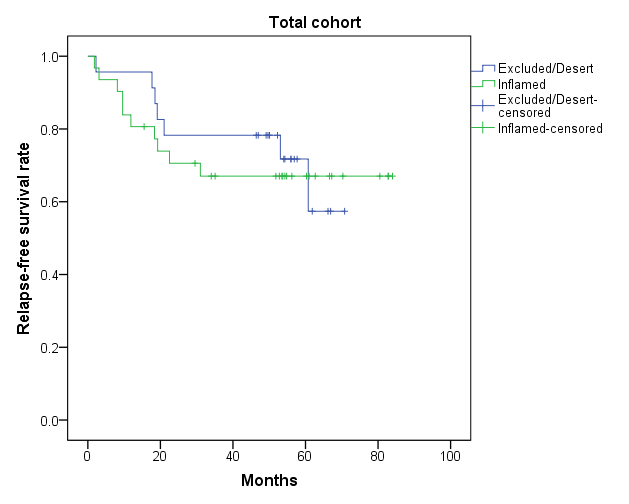

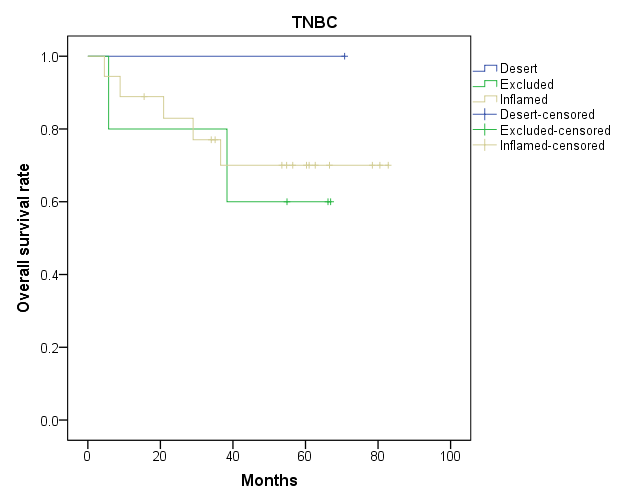

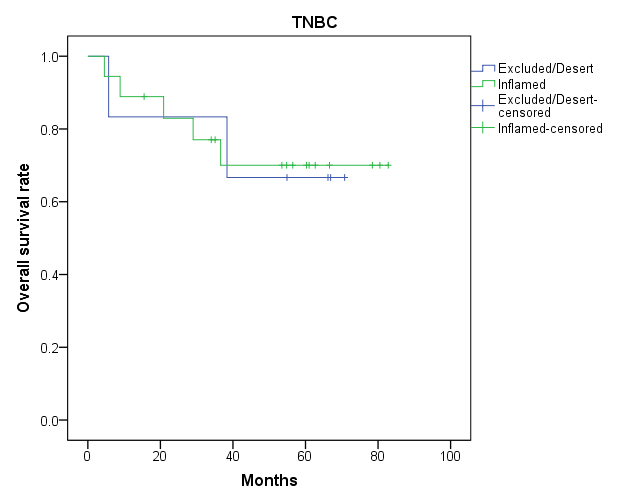


**A**

**B**

**C**

**D**

**E**

**F**

*p=.666*

*p=.440*

*p=.795*

*p=.776*

*p=.777*

*p=.930*

**
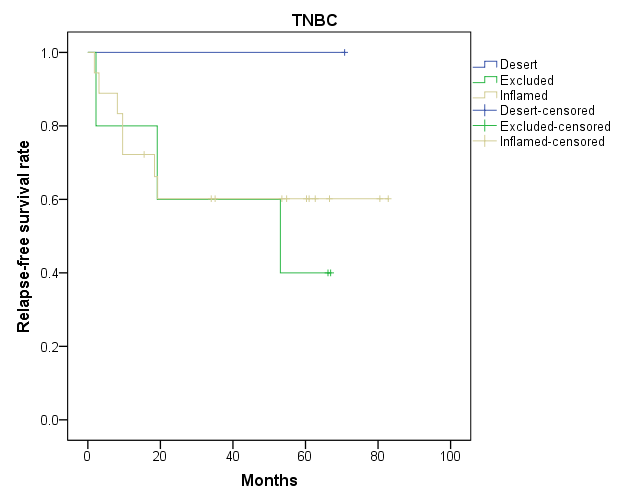

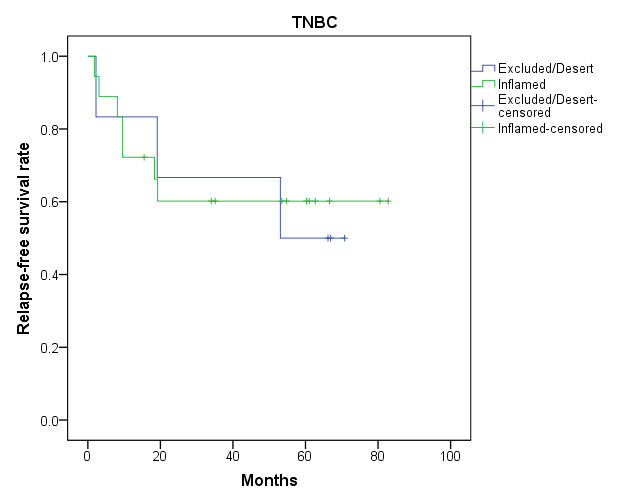
**

**G**

**H**

*p=.630*

*p=.820*
