# Supplementary material for: Proteomic analysis of breast cancer based on immune subtypes
Source: Clin Proteomics. 2024 Feb 29;21:17. doi: 10.1186/s12014-024-09463-y (PMC10905797; doi:10.1186/s12014-024-09463-y)
Supplement: Supplementary file 2 — Additional file 2: Figure S2. The number of identified proteins in the total cohort. Each column states the number of recognized proteins (blue) and NA proteins (red) of each sample among the initially recognized 5,014 proteins in the total cohort. The last column (yellow) states the number of proteins, which have verifiable data for all samples. [file 12014_2024_9463_MOESM2_ESM.docx]

**Additional file 2: Figure S2.** The number of identified proteins in the total cohort. Each column states the number of recognized proteins (blue) and NA proteins (red) of each sample among the initially recognized 5,014 proteins in the total cohort. The last column (yellow) states the number of proteins, which have verifiable data for all samples.

**
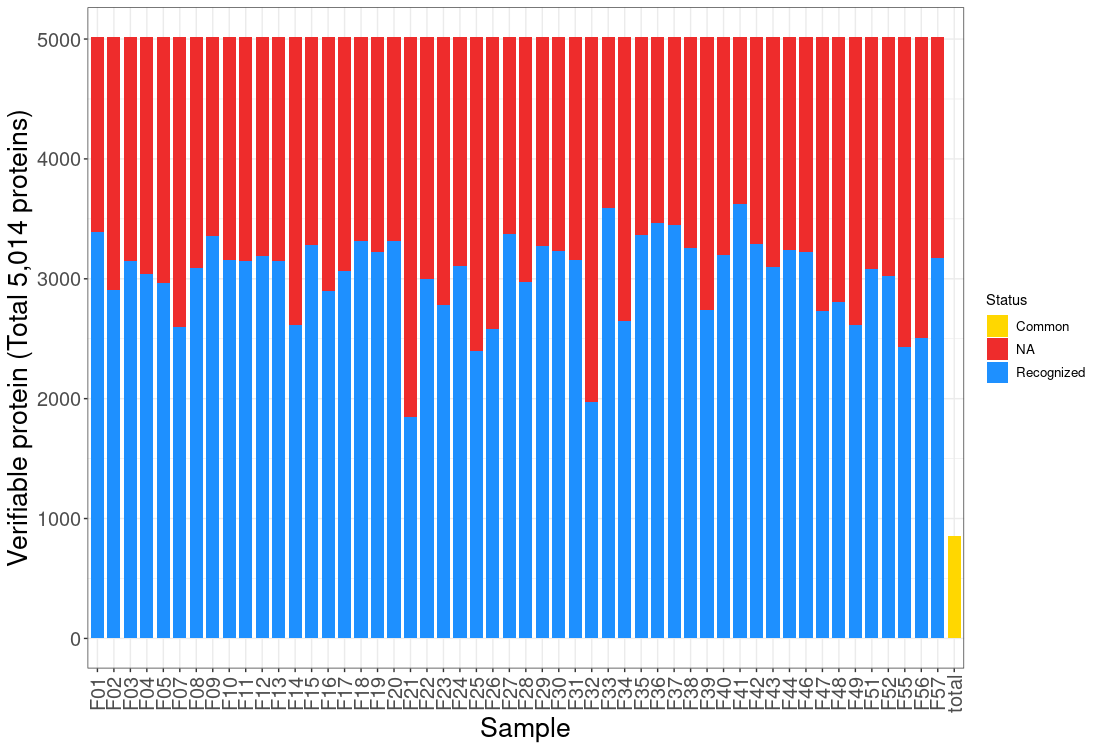
**
