# Supplementary material for: Proteomic analysis of breast cancer based on immune subtypes
Source: Clin Proteomics. 2024 Feb 29;21:17. doi: 10.1186/s12014-024-09463-y (PMC10905797; doi:10.1186/s12014-024-09463-y)
Supplement: Supplementary file 3 — Additional file 3: Table. Gene-enrichment analysis of raw data (5,014 proteins) and confirmed data (855 proteins). [file 12014_2024_9463_MOESM3_ESM.docx]

**Additional file 3: Table.** Gene-enrichment analysis of raw data (5,014 proteins) and confirmed data (855 proteins)

|  | ID | Name | Adjusted *p* |
| --- | --- | --- | --- |
| Raw data |  |  |  |
| Biological Process | GO:0044403 | The biological process involved in the symbiotic interaction. | 1.48E-67 |
|  | GO:0016032 | viral process | 7.09E-67 |
|  | GO:0016071 | mRNA metabolic process | 2.41E-54 |
|  | GO:0006402 | mRNA catabolic process | 5.32E-49 |
|  | GO:0045055 | regulated exocytosis | 4.96E-47 |
| Pathway | 1268678 | Translation | 1.16E-42 |
|  | 1268686 | GTP hydrolysis and joining of the 60S ribosomal subunit | 5.46E-40 |
|  | 1268688 | L13a-mediated translational silencing of Ceruloplasmin expression | 5.46E-40 |
|  | 1268680 | Cap-dependent Translation Initiation | 5.03E-39 |
| Verified data |  |  |  |
| Biological Process | GO:0044403 | The biological process involved in the symbiotic interaction | 9.6E-123 |
|  | GO:0016032 | viral process | 1.2E-121 |
|  | GO:0016071 | mRNA metabolic process | 2.7E-112 |
|  | GO:0016050 | vesicle organization | 9.9E-100 |
|  | GO:0048284 | organelle fusion | 4.9E-99 |
| Pathway | 1457780 | Neutrophil degranulation | 3.72E-56 |
|  | 1269056 | Infectious disease | 9.05E-51 |
|  | 1269688 | Processing of Capped Intron-Containing Pre-mRNA | 7.05E-45 |
|  | 1268678 | Translation | 5.49E-44 |
|  | 1269108 | Influenza Infection | 9.14E-41 |

*GO* Gene Ontology
